# Supplementary material for: Phonon-polaritonic skyrmions: transition from bubble- to Néel-type
Source: Light Sci Appl. 2026 May 18;15:239. doi: 10.1038/s41377-026-02332-3 (PMC13183925; doi:10.1038/s41377-026-02332-3)
Supplement: Supplementary file 1 — Supplementary Information Phonon-polaritonic skyrmions: Transition from bubble- to Néel-type [file 41377_2026_2332_MOESM1_ESM.docx]

**Supplementary Information**

**Phonon-polaritonic skyrmions: Transition from bubble- to Néel-type**

Florian Mangold^†,1^, Enrico Baù^†,2^, Lin Nan^2^, Julian Schwab^1^, Thorsten Gölz^2^, Andrea Mancini^2^, Bettina Frank^1^, Andreas Tittl*^,2^ & Harald Giessen*^,1^

^†^These authors contributed equally to this work

*Corresponding Author

^1^4th Physics Institute, Research Center SCoPE, and Integrated Quantum Science and Technology Center, University of Stuttgart, Germany

^2^Chair in Hybrid Nanosystems, Nano-Institute Munich, Department of Physics, Ludwig-Maximilians Universität München, Germany

**Supplementary Note 1**

For all simulations in this work, the dielectric function of SiC is modeled as a Lorentz oscillator of the following form, with 𝜖_∞_ = 6.6, 𝜔_TO_ = 797 cm^-1^, 𝜔_LO_ =973 cm^-1^, 𝛾 = 1.4 cm^-1^: ^1^

$\varepsilon\left( \omega\right)=\epsilon_{\infty}(1+ \frac{\omega_{LO}^{2}- \omega_{TO}^{2}}{\omega_{TO}^{2}- \omega^{2}-i\omega\gamma})$ (1)

To describe the dispersion of guided modes in bulk polar materials at an interface with air, Maxwell’s equations yield the following expression for the in-plane momentum of SPhPs:

$k_{||}=\frac{\omega}{c}(\frac{\varepsilon(\omega)}{1+\varepsilon(\omega)})$ (2)

In films where the penetration depth exceeds the thickness *t* of the thin polar film, the phonon dispersion splits into a lower and higher energy mode, denoted by $k_{||,1}$ and $k_{||,2}$. The equations in this case are given by:

$\sqrt{\frac{k_{||, 1}^{2}- k_{0}^{2}}{k_{||, 1}^{2}- k_{0}^{2}\varepsilon(\omega)}}=\frac{1}{\varepsilon(\omega)}\tanh(\frac{t}{2}\sqrt{k_{||, 1}^{2}- k_{0}^{2}\varepsilon(\omega}))$ (3)

$\sqrt{\frac{k_{||, 2}^{2}- k_{0}^{2}}{k_{||, 2}^{2}- k_{0}^{2}\varepsilon(\omega)}}=\frac{1}{\varepsilon(\omega)}\coth(\frac{t}{2}\sqrt{k_{||, 2}^{2}- k_{0}^{2}\varepsilon(\omega}))$ (4)

With *α* being the absorption coefficient, the reflectivity of a thin film surrounded with air can be written as:

$R=R_{0}\frac{{(1-e^{-\alpha t})}^{2}+4e^{-\alpha t}\sin^{2} \beta}{{(1-e^{-\alpha t})}^{2}+4e^{-\alpha t}\sin^{2} (\beta+\varphi_{r})}$ (5)

where *R_0_* is the reflectivity of a semi-infinite slab and can be written as:

$R_{0}=\frac{{(1-n)}^{2}+k^{2}}{{(1+n)}^{2}+k^{2}}$ (6)

and $\varphi_{r}$ the phase change upon reflection, which is written as:

$\varphi_{r}= \tan^{-1} (\frac{-2k}{1-n^{2}-k^{2}})$ (7)

with *n* and *k* being the real and imaginary part of the refractive index respectively.

**
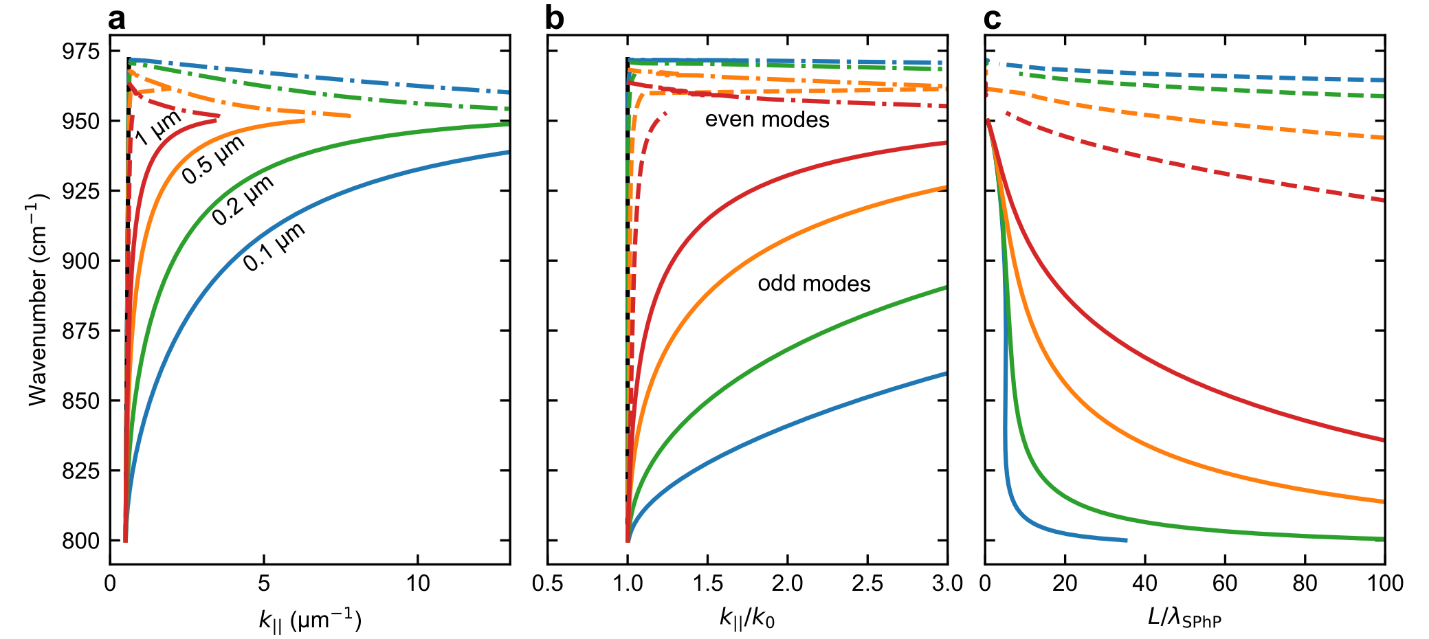
**

**Figure S1. Dispersion and properties of SPhPs in SiC.** **a** Simulated SPhP wavevector *k_||_*, **b** ratio between in-plane *k_||_* and free space *k_0_* momentum and **c** excitation wavenumber vs. propagation length *L/λ*_SPhP_ calculated for different film thicknesses. Full curve are odd modes, while dashed curves are even modes. Black full curve displays the light line where *k_||_* = *k_0_*.


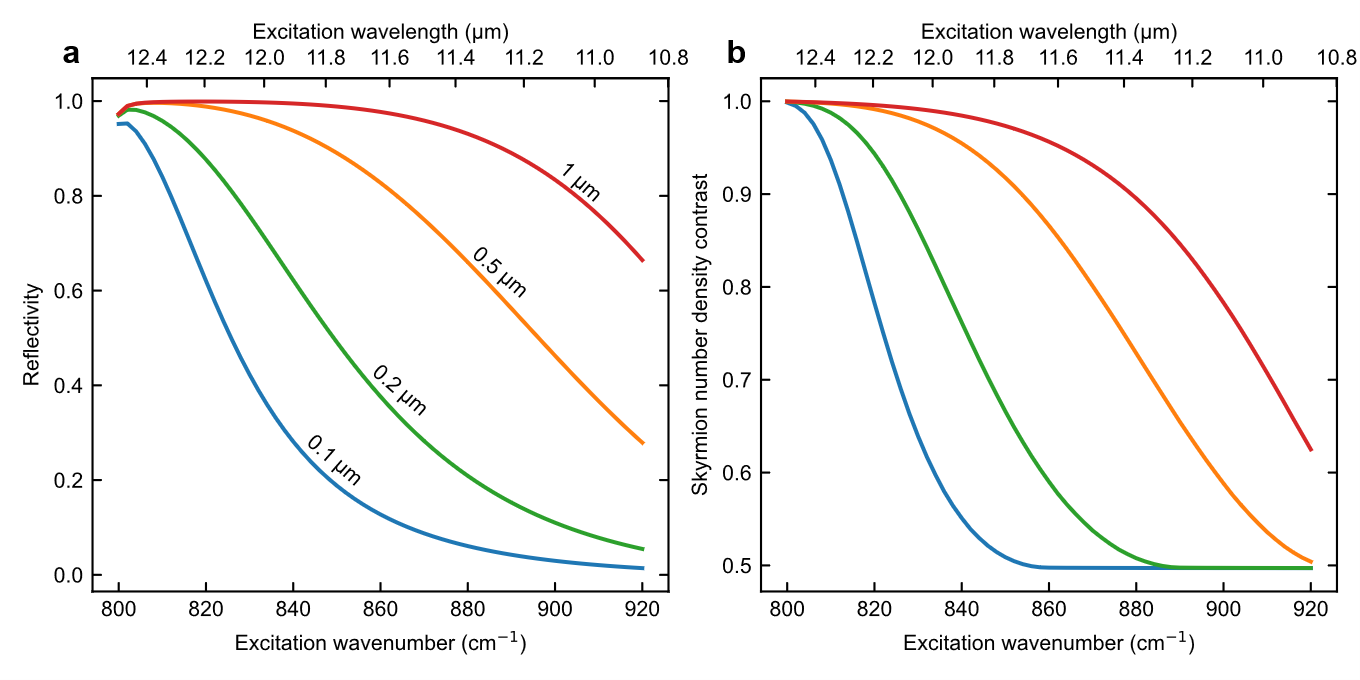


**Figure S2. a** Calculated reflectivity and **b** skyrmion number density contrast vs. excitation wavenumber of SiC films for different film thicknesses.


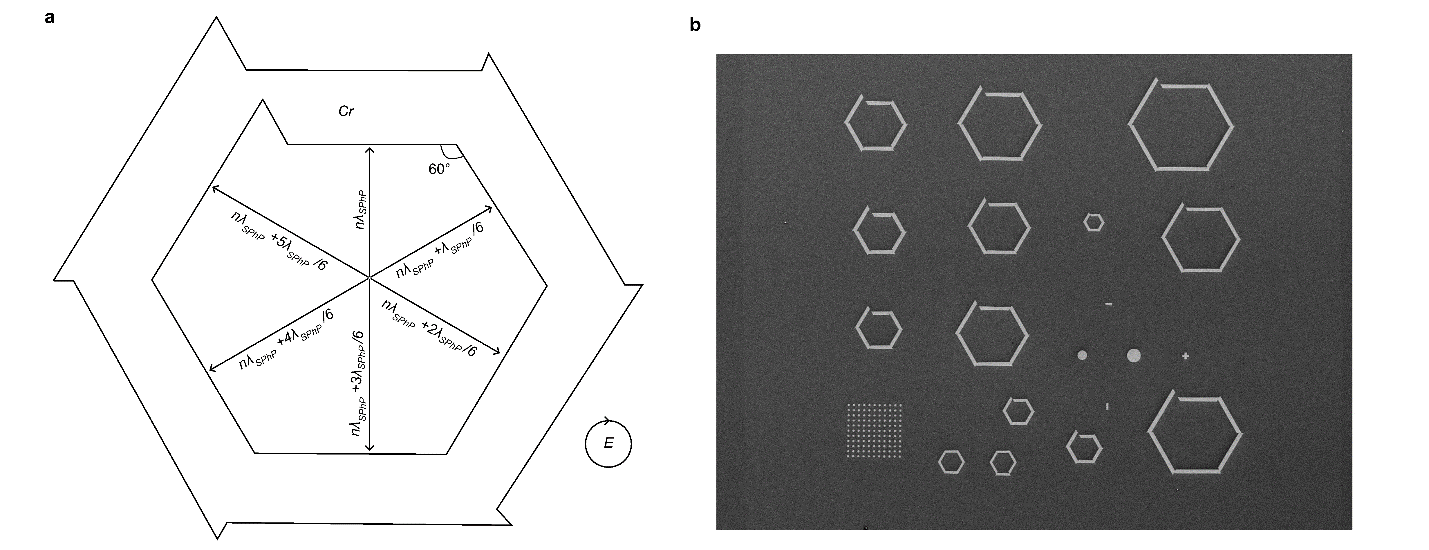


**Figure S3. Chromium (Cr) structures used to launch SPhPs and generate phonon-polaritonic skyrmion lattices. a** Schematic of the hexagonal launcher geometry. The distance between the centre of the structure *R(n,m)* and each chromium edge follows the rule *R(n,m)=nλ*_SPhP_*+mλ*_SPhP_*/6* where n and m are positive integers. The number of skyrmions generated in such a structure is equal to *N*_skyrmions_ *= 3n^2^-3n+1*. Additionally, starting from the first edge, each side of the hexagonal structure is consecutively offset by an additional factor of *λ*/6 (increasing m by 1) in order to compensate for the phase delay occurring due to the circularly polarized light. This design ensures constructive interference of the circularly polarized excitation. All edges have the same width (around 3.5 μm) and height (around 30 nm). All measurements in this study were conducted on structures with *n = 3*. Values of *n < 2* produce boundary effects, while *n > 4* reduce the signal-to-noise ratio. Radially polarized light would allow continuous wavelength tuning, although we were not able to investigate this experimentally. **b** SEM image of fabricated structures on a 200 nm thick SiC membrane.


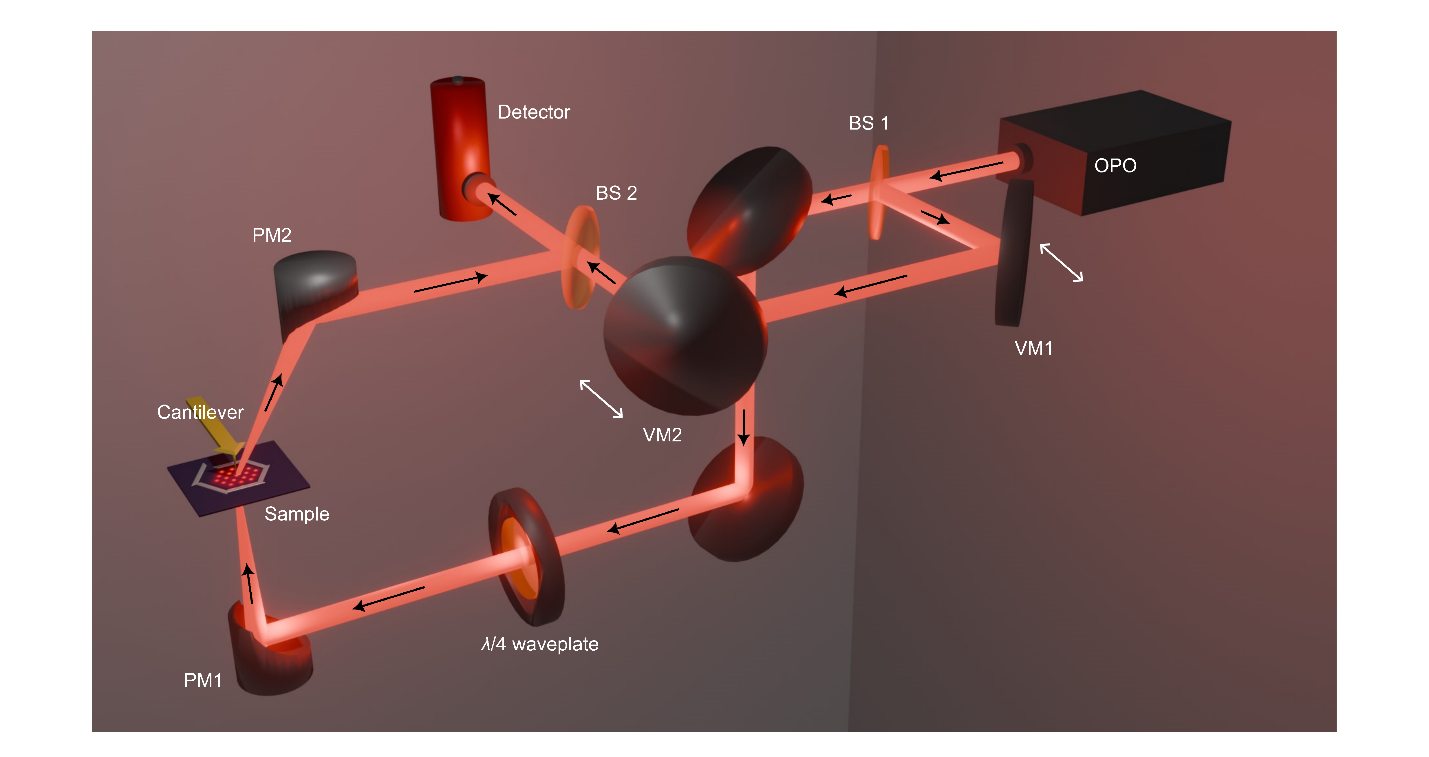


**Figure S4. Transmission mode s-SNOM operated in tapping mode.** A laser beam generated by an optical parametric oscillator (OPO) hits a beamsplitter (BS1), after which one half travels through a *λ*/4 waveplate to achieve circular polarization. The circularly polarized light is then loosely focused onto the sample and the apex of a sharp AFM tip by a parabolic mirror (PM 1). The backscattered light from the tip is then collected by a second parabolic mirror (PM 2) above the sample and interferes with a reference beam at the second beamsplitter (BS2). The superposition is detected by an MCT-detector. The reference beam is modulated by a pair of vibrating reference mirrors (VM1/VM2) to decouple near-field amplitude and phase (pseudo-heterodyne detection scheme).

**Supplementary Note 2**

The laser illumination creates charge excitations at the edges of the coupling structure, which launches the surface phonon polariton wave that propagate on the SiC surface. The electric field of the surface phonon polariton launched from a single boundary of the coupling structure is given by

$\boldsymbol{E}\left( \boldsymbol{r} \right)=E_{L}e^{-k_{z}z} e^{-i\omega_{0}t} \int\frac{ik_{z}\hat{\boldsymbol{r}_{\boldsymbol{s}}} -k_{||} \hat{\boldsymbol{z}}}{k_{0}}\left( \hat{\boldsymbol{e}}\cdot\hat{\boldsymbol{n}} \right)H_{0}^{\left( 1 \right)}\left( k_{||}r_{s} \right) d^{2}\boldsymbol{r}^{'},$ (10)where $\hat{\boldsymbol{n}}$ is the unit normal vector of the boundary and $\hat{\boldsymbol{e}}$ is the polarization vector of the incident light^2^. $k_{||}$ and $k_{z}$ denote the out-of-plane and in-plane components of the SPhP electric field wave number, which are calculated using eqs. (3) and (4) from the vacuum wave number $k_{0}=2\pi/\lambda_{0}$ of the excitation laser with wavelength $\lambda_{0}$ and angular frequency $\omega_{0}$. $\boldsymbol{r}^{\boldsymbol{'}}$ are the points along the boundary, where the incident light excites the surface wave and $\boldsymbol{r}_{\boldsymbol{s}}=\boldsymbol{r}-\boldsymbol{r'}$ is the distance to these course points, with  $\hat{\boldsymbol{r}_{\boldsymbol{s}}}=\boldsymbol{r}_{\boldsymbol{s}}/r_{s}$. Furthermore, $H_{0}^{\left( 1 \right)}(k_{||}r_{s})$ is the Hankel function of the first kind involving the Bessel functions $J_{0}$ and $iY_{0}.$Here, the pulsed shape of the excitation is neglected, since in our system the spatial width of the SPhP wave is much longer than its propagation distance and the size of the coupling structure.

An numerical evaluation of the integral is obtained by distributing a finite amount of Huygens sources along the boundary, with a spacing smaller than $\lambda_{\mathrm{SPhP}}/5$. The integral is then approximated by the sum of these Huygens wavelets, where each wavelet is of the form of the integrand of eq. (10):

$E_{\mathrm{wavelet}}\left( r \right)=E_{L}e^{-k_{z}z} e^{-i\omega_{0}t}\frac{ik_{z}\hat{r_{s}} -k_{||} \hat{z}}{k_{0}}\left( \hat{e}\cdot\hat{n} \right)H_{0}^{\left( 1 \right)}\left( k_{||}r_{s} \right)$(11)

In the case of coupling structures with multiple boundaries, the Huygens sources are simply distributed along all boundaries with the same spacing distance and the sum of all Huygens wavelets yields the total SPhP electric field. This procedure follows the approach described in Davis et al.^2^, which provides an efficient and accurate method for modeling surface wave excitation.

**Supplementary Note 3**

Before measuring the sample, the alignment of the SNOM should be thoroughly checked. In addition to standard procedures, we ensured that the illumination was homogeneous, and that the alignment of the bottom parabolic mirror was correct so that the light struck the sample at normal incidence (see **Fig. S11**). The acquired data was mostly demodulated at the third order; however, for larger wavenumbers where the signal to noise ratio was smaller, we used the second demodulation order. The data analysis was performed in Python and can be divided into two steps: the calculation of the complex out-of-plane electric field component, *E_z_* (**Fig. S7**), and the skyrmion data analysis and characterization (**Fig. S8**). Static information about the intensity distribution of the out-of-plane component of the SPhP electric field is provided using SNOM images.^1,3,4^ The pseudoheterodyne measurement yields amplitude *A_z_* and phase $\theta_{z}$ of the out-of-plane field, which is then given by $E_{z}=A_{z}\exp\left( i\theta_{z} \right)$. The raw data needs to be spatially Fourier filtered to remove unwanted contributions, leaving only the SPhP with $k=k_{\mathrm{SPhP}}$. The resolution of the Fourier transform is increased using zero padding, where the measurement area is increased and filled with a vanishing field ($E_{z}= 0$) before the Fourier transform. After the Fourier transform, the data is filtered with a radial filter around
$k=k_{\mathrm{SPhP}}$ and then transformed back using the inverse Fourier transform. After that, the field is cropped to its original size. Subsequently, the in-plane components of the field can be calculated by using Maxwell’s equations and the TM polarization of evanescent surface waves. The resulting fields are

$$E_{x}=-\frac{\gamma}{k_{\mathrm{SPhP}}^{2}}\frac{dE_{z}}{dx}, and$$

$$E_{y}=-\frac{\gamma}{k_{\mathrm{SPhP}}^{2}}\frac{dE_{z}}{dy},$$

where $\gamma^{2}=k_{\mathrm{SPhP}}^{2}-k_{0}^{2}$ is the SPhP wavevector in $z$-direction ($E_{\mathrm{SPhP}}\propto exp(-\gamma z)$).

The raw data and calculated properties for all wavenumbers are displayed in **Fig. S5**. The experimental data analyzed with different figures of merit (SNDC, domain wall steepness, and domain wall size) for selective wavenumbers covering the transition from bubble- to Néel-type skyrmions is shown in **Fig. S9**. Additionally, **Fig. S10** presents the domain wall steepness for all measured wavenumbers, alongside its inverse, which is used to avoid divergence towards infinity for wavenumbers close to 800 cm⁻¹.


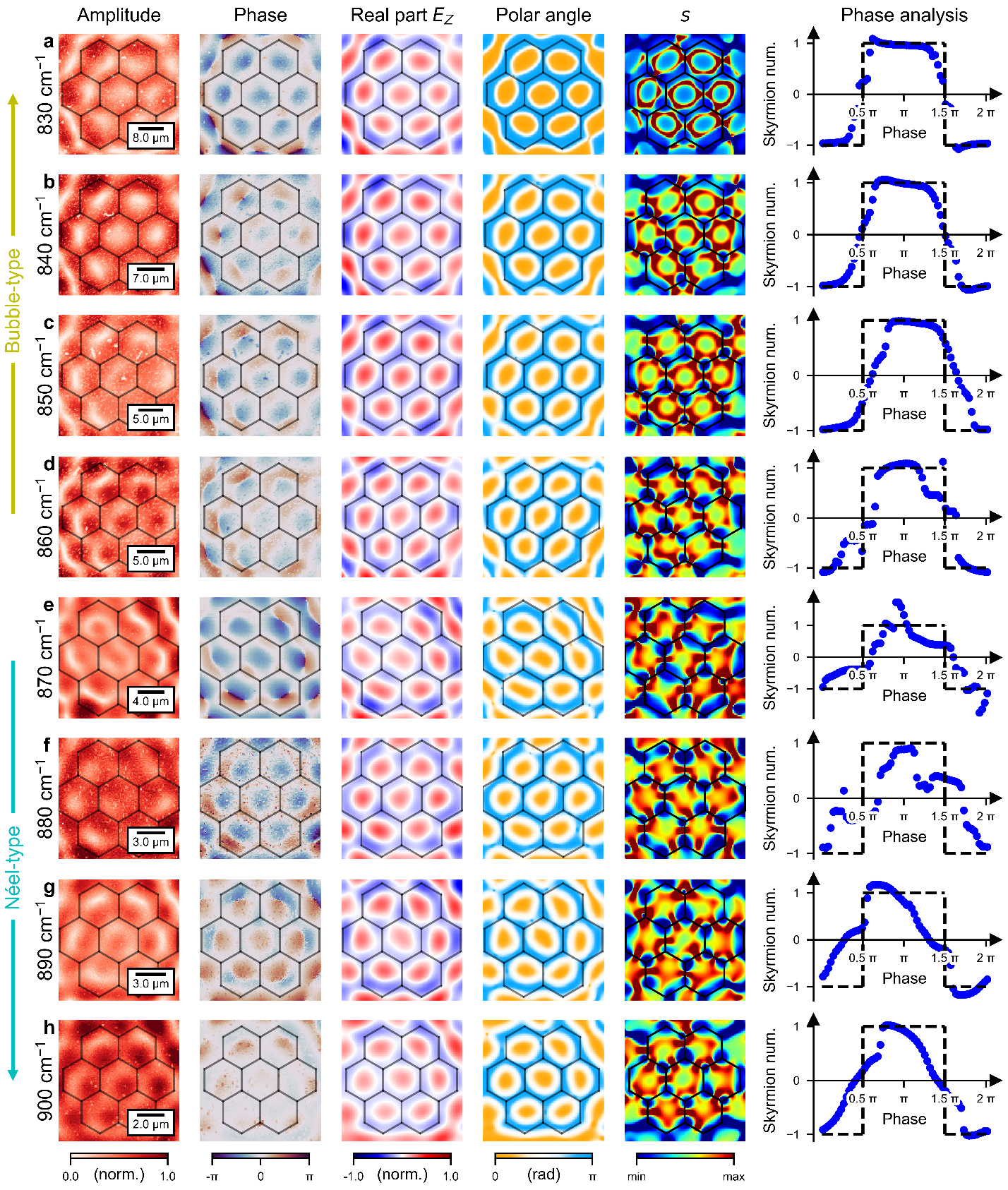


**Figure S5. Experimental measurements and tunability of phonon-polariton optical skyrmions. a-h** From left to right: measured near-field optical amplitude *s_3_*, near-field optical phase *φ_3_*, Fourier-filtered real part of the out-of-plane electric field *E_z_,* Fourier-filtered polar angle *θ,* skyrmion number density (SND) and phase stability of the skyrmion number. Measurements were conducted between 830-900 cm^-1^.


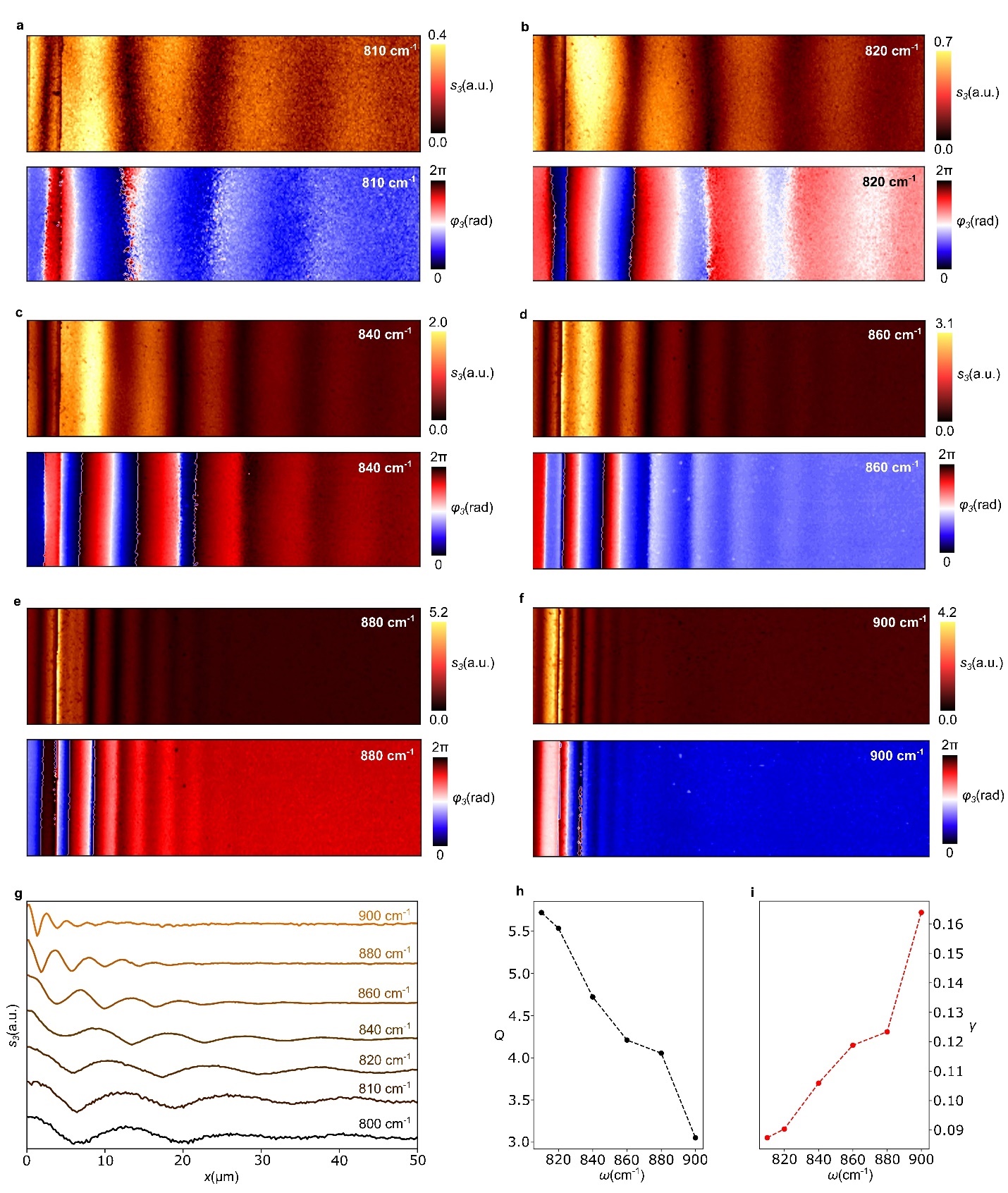


**Figure S6. Experimental measurements of SPhP propagation. a-f** Near-field amplitude *s_3_* and phase *φ_3_* images showing SPhPs launched from a chromium edge at different incident wavenumbers ranging from 800 to 900 cm^-1^. **g** Profiles extracted from a-f showing SPhP propagation along a single line of scanning. **h, i** Quality factor *Q* and damping factor γ extracted from g. The curves are hereby fitted by a function of the form
$A=A_{0}sin(kx)e^{-i\gamma x}$ and the quality factor is obtained via $Q=\frac{1}{2\gamma}$.

**
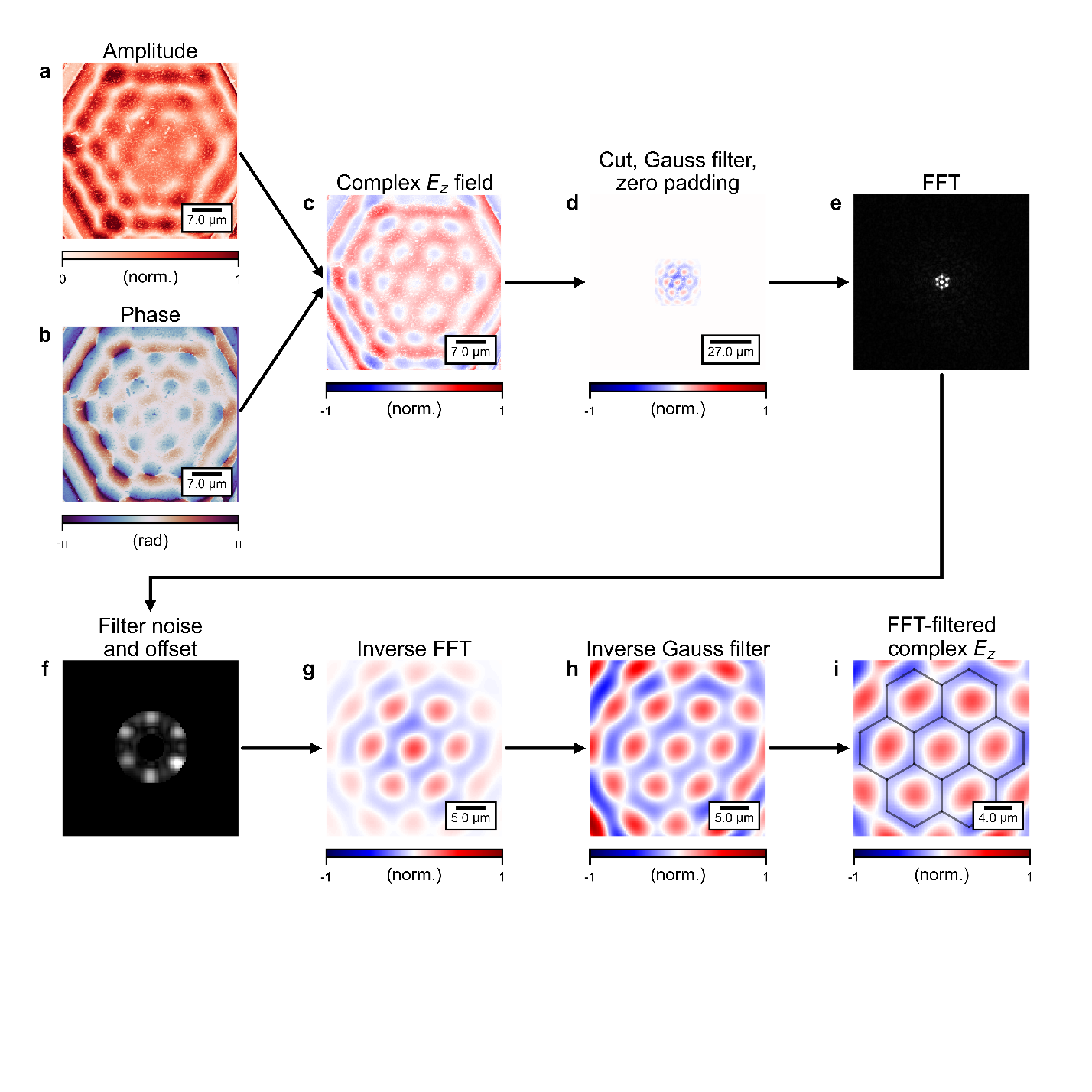
**

**Figure S7. Workflow of SNOM data analysis.** The measured amplitude (**a**) and phase (**b**) are used to compute the complex out-of-plane electric field, *E_z_* (**c**). The region of interest is selected and to enhanced the resolution zero padding is used. A Gaussian filter is applied (**d**) to minimize boundary effects caused by the periodic nature of the fast Fourier transform (FFT) (**e**). Following this, both low-pass and high-pass filters are applied to eliminate noise and any offset that could not be avoided during the SNOM measurement (**f**). Then the inverse FFT is performed (**g**) and the inverse Gaussian filter is applied (**h**). In the final step, skyrmions are identified, marked, and isolated from the surrounding area to obtain the FFT-filtered complex *E_z_*, which is then used for further investigation of skyrmionic properties (**i**).

#
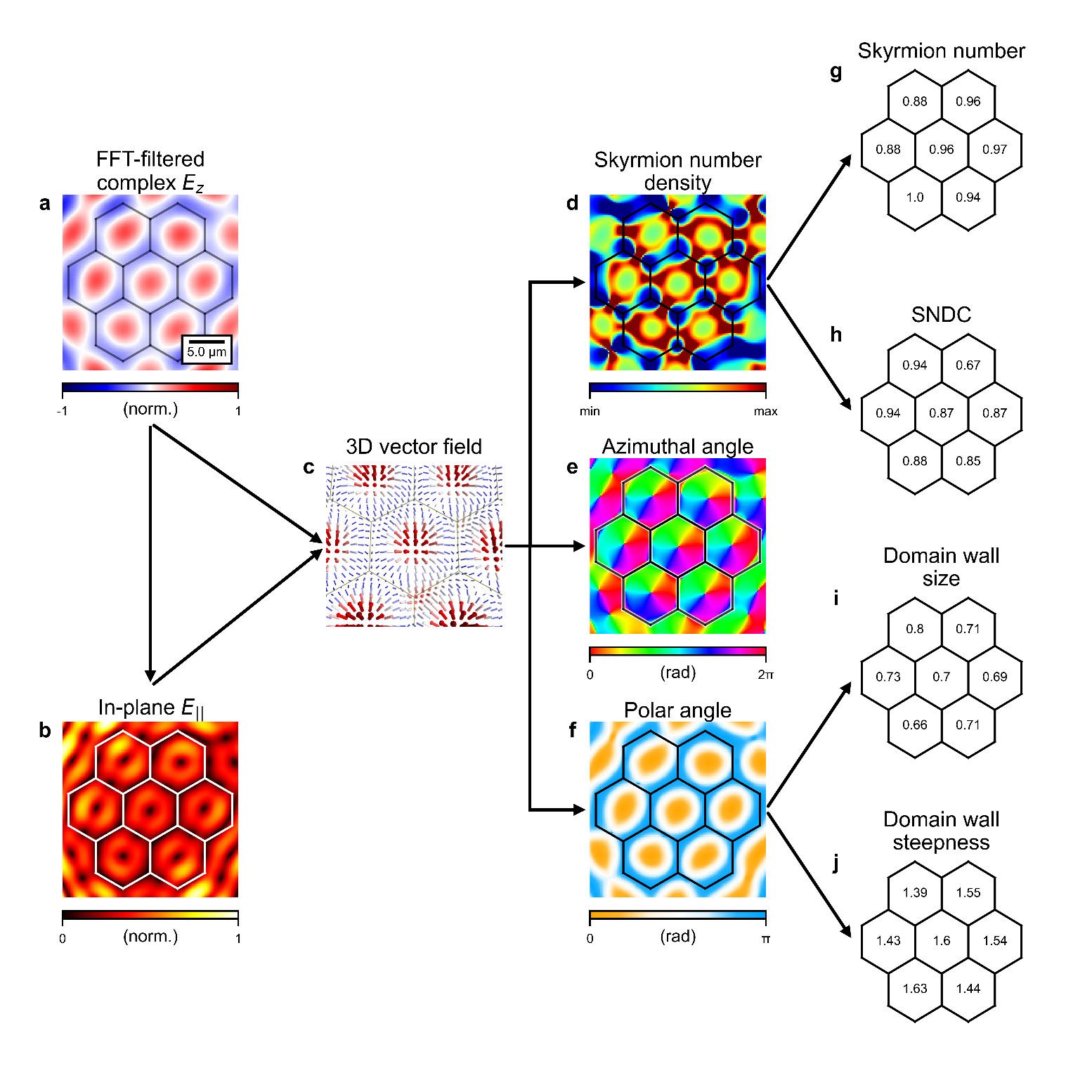


# Figure S8. Workflow of skyrmion data analysis and characterization. The previously calculated FFT-filtered complex out-of-plane electric field *E_z_* (a) is used to derive the in-plane electric field *E_||_* (b)^4^. By combining the in-plane and out-of-plane components, a 3D vector field is generated (c). Using equation (1) from the main manuscript, the skyrmion number density (SND) (d) is computed and integrated to determine the skyrmion number (g). Additionally, the skyrmion number density contrast (SNDC) is calculated (h). The 3D vector field can also be used to extract the azimuthal (e) and polar angles (f), the latter being employed to derive the newly introduced figures of merit (FOM): domain wall size (i) and domain wall steepness (j).

**
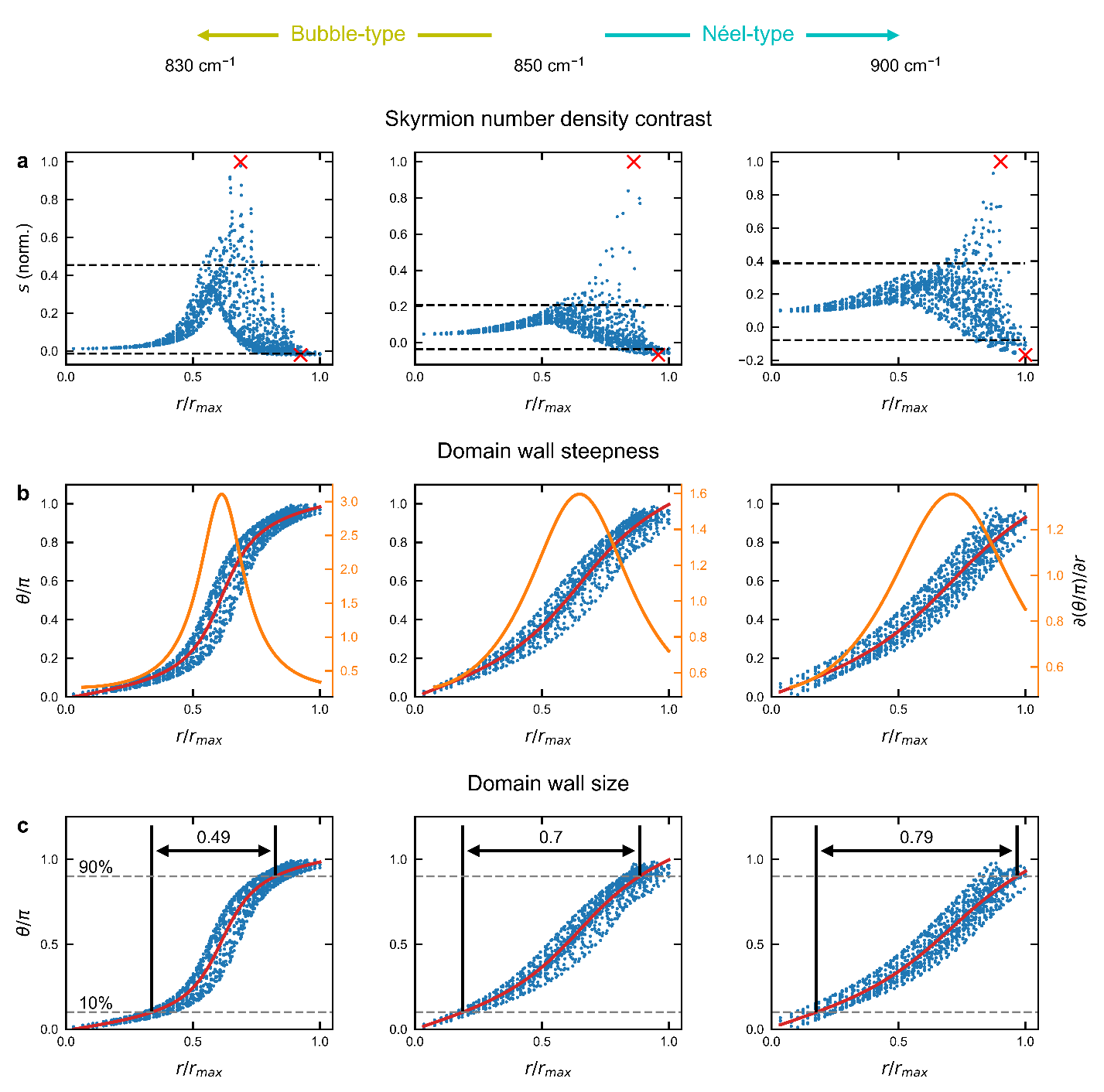
**

**Figure S9. Experimental data analyzed with different figures of merit (FOMs) for various wavenumbers, covering the transition from bubble- to Néel-type skyrmions.** To calculate the skyrmion number density contrast (SNDC) (**a**), the original method used the minimum and maximum values of the SND (marked by the red cross). However, to reduce the influence of potential outliers, the 5th and 95th percentiles (black dashed lines) of the SND were used to determine the SNDC. The domain wall steepness and size are calculated by sorting the polar angle data within each unit cell of the skyrmion lattice based on their distance to the center and fitting the data (red line) using $\tan(E_{\theta})=a\cdot\tan(r/r_{max}\cdot\pi)$, where 𝑎 is the fitting parameter controlling the steepness of the curve. This formula is derived from the mathematical framework of a plane wave. The derivative of the fit (orange line) is used to identify the maximum steepness, which represents the domain wall steepness (**b**). The domain wall size (**c**) is defined as the distance between the 10% and 90% thresholds of the fit function (dashed gray line). The domain wall size is stated in the figure.

**
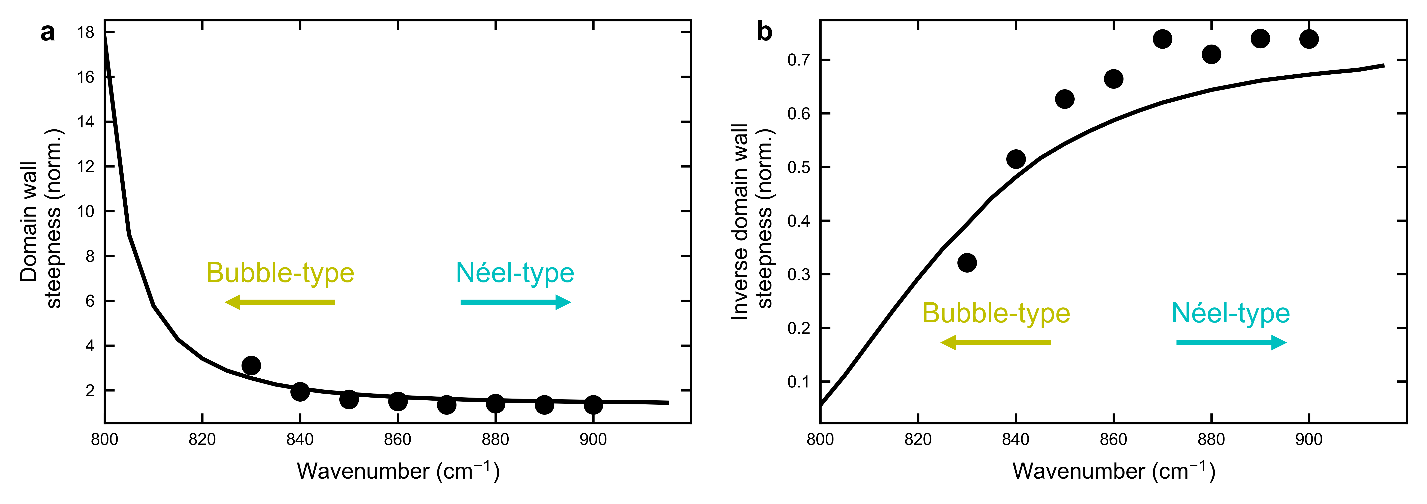
Figure S10. Comparison of the domain wall steepness with its inverse.** Measured data is represented by points and simulated values are plotted as a line. In (**a**), the steepness approaches infinity for small wavenumbers, making it less suitable as a figure of merit (FOM). By taking its inverse (**b**), this issue is mitigated and the deviations between experimental and simulated data becomes more apparent.


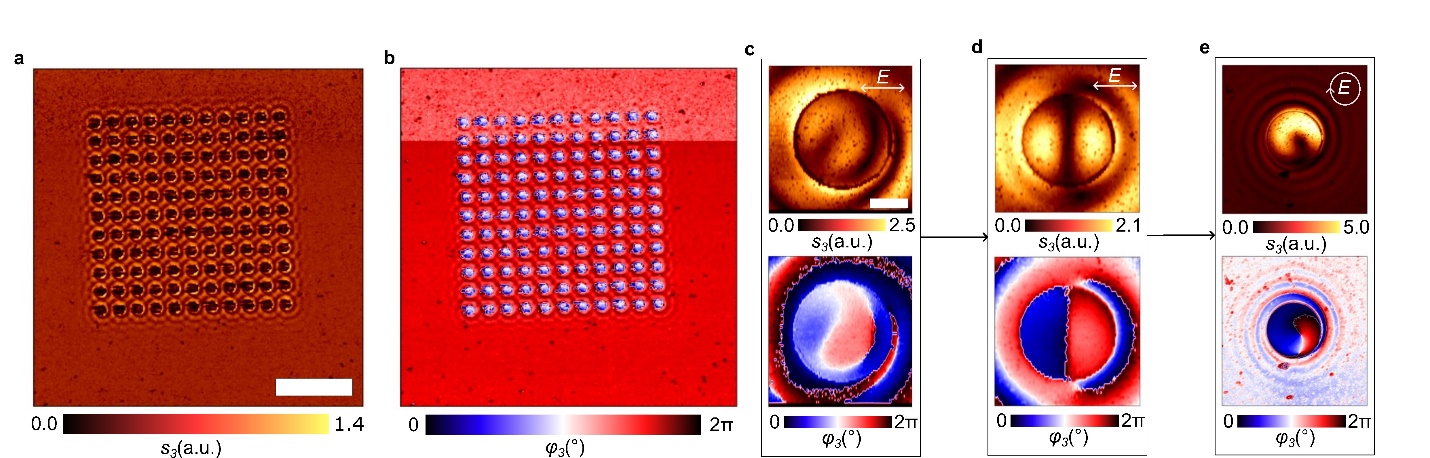


**Figure S11. Experimental measurements of beam spot size and alignment procedure. a, b** Near-field amplitude *s_3_* and phase *φ_3_* images of an array of chromium resonators (radius 1 μm) measured with synchronized bottom illumination. The uniformity of the signal proves that the beam spot size used for measurements is much larger than the illuminated chromium structures used to excite optical skyrmion lattices. Scale bar 25 μm. **c, d** Near-field amplitude *s_3_* and phase *φ_3_* images of a single chromium disk (radius 10 μm) using linear polarization, measured before c and after d proper alignment of the bottom parabolic mirror. If the incident beam is properly aligned, linear polarization yields a clear dipole in both amplitude and phase, as seen in d. Scale bar 2.5 μm. **e** Same as d, but using circular polarization. The chromium disk launch SPhPs which form a vortex around it.


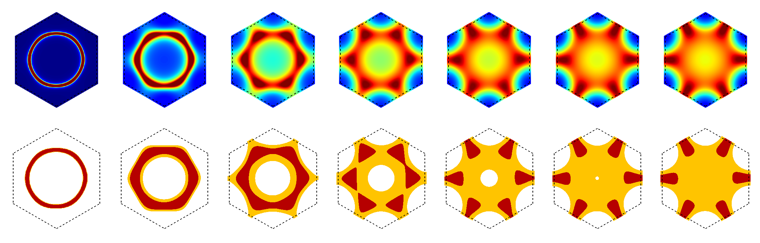


**Figure S12. Transition from bubble- to Néel-type skyrmions.** Comparison between simulation (top) and simplified representation (bottom) of the skyrmion number density from 800 cm^-1^ to 920 cm^-1^ in 20 cm^-1^ steps. In the simplified representation, the skyrmion number density is mapped onto three discrete levels to facilitate visual identification of the continuous transition: regions of highest skyrmion number density (> 0.4 a.u.) are shown in brown, intermediate values in yellow (0.4-0.2 a.u.), and low-density regions in white (< 0.2 a.u.).

References

1. Mancini, A. *et al.* Near-Field Retrieval of the Surface Phonon Polariton Dispersion in Free-Standing Silicon Carbide Thin Films. *ACS Photonics* **9,** 3696–3704; 10.1021/acsphotonics.2c01270 (2022).

2. Davis, T. J. *et al.* Subfemtosecond and Nanometer Plasmon Dynamics with Photoelectron Microscopy: Theory and Efficient Simulations. *ACS Photonics* **4,** 2461–2469; 10.1021/acsphotonics.7b00676 (2017).

3. Ocelic, N., Huber, A. & Hillenbrand, R. Pseudoheterodyne detection for background-free near-field spectroscopy. *Applied Physics Letters* **89**; 10.1063/1.2348781 (2006).

4. Tsesses, S. *et al.* Optical skyrmion lattice in evanescent electromagnetic fields. *Science (New York, N.Y.)* **361,** 993–996; 10.1126/science.aau0227 (2018).
